# Monocular enucleation alters retinal waves in the surviving eye

**DOI:** 10.1186/s13064-018-0101-1

**Published:** 2018-03-24

**Authors:** Samuel Wilson Failor, Arash Ng, Hwai-Jong Cheng

**Affiliations:** 1 Center for Neuroscience, University of California, Davis, 1544 Newton Court, Davis, CA 95618 USA; 2 Department of Neurobiology, Physiology, and Behavior, University of California, Davis, One Shields Avenue, Davis, CA 95616 USA; 3 Department of Pathology and Laboratory Medicine, University of California, Davis, One Shields Avenue, Davis, CA 95616 USA; 4 Wolfson Institute for Biomedical Research, University College London, Gower Street, London, WC1E 6BT UK

## Abstract

**Background:**

Activity in neurons drives afferent competition that is critical for the refinement of nascent neural circuits. In ferrets, when an eye is lost in early development, surviving retinogeniculate afferents from the spared eye spread across the thalamus in a manner that is dependent on spontaneous retinal activity. However, how this spontaneous activity, also known as retinal waves, might dynamically regulate afferent terminal targeting remains unknown.

**Methods:**

We recorded retinal waves from retinae ex vivo using multi-electrode arrays. Retinae came from ferrets who were binocular or who had one eye surgically removed at birth. Linear mixed effects models were used to investigate the effects of early monocular enucleation on retinal wave activity.

**Results:**

When an eye is removed at birth, spontaneous bursts of action potentials by retinal ganglion cells (RGCs) in the surviving eye are shorter in duration. The shortening of RGC burst duration results in decreased pairwise RGC correlations across the retina and is associated with the retinal wave-dependent spread of retinogeniculate afferents previously reported in enucleates.

**Conclusion:**

Our findings show that removal of the competing eye modulates retinal waves and could underlie the dynamic regulation of competition-based refinement during retinogeniculate development.

## Background

Developing nascent neural circuitry undergoes modifications in an activity-dependent manner [1–6]. Neural activity that is essential for early stages of visual system development originates from spontaneous processes [1, 4–6] and appears to facilitate circuit refinement by driving Hebbian-like competition for synaptic partners between innervating neurons [7–9]. This activity-dependent refinement results in the precise mapping of sensory areas, for example by establishing eye-specific laminae and fine-scale retinotopy across visual areas [1, 4].

Patterned spontaneous retinal activity (i.e. retinal waves) occurs primarily during periods of functional blindness [10–13] and is characterized by periodically occurring domains of retinal ganglion cell (RGC) activity that slowly propagate across the retina in a wave-like fashion. This spatiotemporal feature of retinal waves leads to a high level of correlated activity between neighboring RGCs and very little correlated activity between RGCs that are distant from each other. Retinal waves have been shown to play a critical role in the establishment of eye-specific laminae in the dorsal lateral geniculate nucleus (dLGN) [7, 8, 14–20], as well as fine-scale retinotopy [8, 21] and receptive field size [8, 22, 23] in the dLGN and superior colliculus. For example, when an eye is lost early in development, retinogeniculate afferents from the surviving eye spread across the dLGN in a retinal wave-dependent manner [8]. This study demonstrated that retinal waves drive both inter-eye [7] and intra-eye competition for synaptic space in the dLGN. However, it remains unclear how retinal waves might facilitate retinogeniculate expansion. One possibility is that the loss of the competing eye alters retinal waves to guide this process.

Here we show that in ferrets when we surgically remove a competing eye, retinal waves in the surviving eye were altered. Primarily, retinal wave associated bursts of action potentials by retinal ganglion cells in the surviving eye were shorter as were the number of spikes contained in these bursts. The shortening of bursts also decreased levels of pairwise RGC correlation. Thus, a significant reduction in levels of correlated RGC activity during retinal waves is associated with removal of the competing eye.

Based on these data, we propose a model where the presence of the competing eye reduces intra-eye competition for synaptic space in the dLGN by increasing correlated RGC activity, which facilitates the formation of eye-specific laminae during inter-eye competition. Conversely, the absence of the competing eye promotes the expansion of retinogeniculate laminae by reducing pairwise RGC correlations and increasing intra-eye competition. In this way, adjustments to the duration of RGC bursts during retinal waves could dynamically optimise competition-based retinogeniculate refinement during the establishment of eye-specific laminae.

## Methods

### Animals

Time-pregnant fitch-coat ferrets were received at mid to late gestation, giving birth 2–3 weeks later (Marshall BioResources, NY, USA; RRID:SCR_015489). Food and water were provided ad libitum. All procedures were authorized by the University of California, Davis (RRID:SCR_012713) Institutional Animal Care and Use Committee (IACUC) and performed in accordance with national and international standards for humane animal research as set forth by the National Institutes of Health (RRID:SCR_011417), Institute of Laboratory Animal Research (RRID:SCR_006872), USDA (RRID:SCR_011486), and Assessment and Accreditation of Laboratory Animal Care, International (RRID:SCR_015496).

### Monocular enucleation

Neonatal ferrets of either sex were anesthetized with isoflurane at P1. After topical lidocaine was applied, the eyelids of one eye were separated, and the muscles and connective tissue of the eyeball were blunt dissected. Hemostats were used to clamp the optic nerve after which it was severed and the eyeball removed. Antibiotic ointment was applied to the orbit, and sterile gelfoam was inserted to stem any subsequent bleeding. A liquid suture was applied to seal the eyelids. Before the animal fully awakened a single dose of buprenex was administered intramuscularly (0.02 mg/kg) as a postoperative analgesic. The monocular enucleation procedure typically took under 5 min. Age-matched littermates served as controls.

### Multielectrode array recordings

Ferrets were euthanized with a lethal dose of pentobarbital (0.1–0.2 ml) via an inter-peritoneal injection. An eye was enucleated, and the retina was removed and stored in ice-cold buffered and oxygenated media (M7278, Sigma-Aldrich, USA; RRID:SCR_008988). A piece of the retina was placed RGC side down onto a 60-channel MEA (MEA2100 System, Multi-Channel Systems, Germany; RRID:SCR_014809), and held in place with a piece of dialysis membrane (Spectrapore 132,130, Spectrum Labs, USA; RRID:SCR_015488). The tissue was superfused with buffered media at 1–2 ml/min at 34 or 37 °C. The array electrodes were 30 μm in diameter and arranged in an 8 × 8 rectilinear grid with an interelectrode spacing of 200 μm. At this distance, the signal for a given cell appeared on only one electrode, so each isolated cell was assigned the spatial coordinates of the electrode on which it was recorded. Analog data were acquired at 20 kHz per channel simultaneously from each electrode. After the retina had been placed on the MEA, the tissue was allowed to acclimate for at least 45 min. When retinal waves appeared stable, recordings were performed for 20 min.

### Spike identification

Raw data were digitally filtered with a 125-Hz high-pass filter (four-pole Butterworth) for sorting spike events. A threshold of six STD was set for each channel and 1 ms of data before, and 4 ms after a threshold-crossing event were stored for each negative-slope event. These candidate spike waveforms were then sorted with Offline Sorter (Plexon, USA; RRID:SCR_000012) using the first three principal components of the spike waveforms. Coincident events within 0.5 ms of each other that were detected on at least 90% of the channels were attributed to perfusion noise and removed. Clusters were first identified using an EM cluster algorithm [24] then manually edited for clustering errors. Typically, each electrode recorded the activity of one to three cells.

### Analysis of RGC burst properties

RGC bursts were identified as previously described [25]. All burst analyses were carried out using custom scripts written in Matlab (Mathworks, USA; RRID:SCR_001622). The beginning of a burst was defined as the point in an RGC spike train when the inter-spike interval (ISI) was less than 0.1 s. Subsequent spikes with ISIs less than 1 s were included in the burst, whereas an ISI of greater than 1 s denoted the end of the burst. If two bursts occurred within 5 s of each other, they were merged.

The properties of bursts identified by this algorithm were then averaged for each cell. Firing rate, burst duration, the number of spikes within a burst, the percentage of spikes within bursts, burst frequency, burst ISI, the percentage of burst time above 10 spikes/s, the percentage of bursts in waves, and bursts per wave were all quantified.

### Analysis of wave properties

Retinal waves were identified in a similar way to that previously described [26]. All wave analyses were carried out using custom scripts written in Matlab (Mathworks, USA; RRID:SCR_001622). MEA recordings were divided into 1 s time bins. The beginning of a retinal wave was defined as the time bin when greater than 5% of all cells were bursting and considered over when less than 2.5% were bursting.

The position of a wave over time was the center of mass of the cells participating in the wave in each time bin. Wave speed was defined as the average change in wave position over time. Wave spread was defined as the average percentage of new electrodes that detected bursting cells in each time bin. Waves that lasted for less than 3 s were not included in analyses of wave speed or spread.

The size of a wave was defined as the average percentage of electrodes that detected bursting cells across the duration of a wave.

### Correlation analysis

Correlation analyses were carried out using custom scripts written in Matlab (Mathworks, USA; RRID:SCR_001622). Pairwise correlations between RGC spike trains were measured by calculating the spike time tiling coefficient [27] (STTC), which is bounded and insensitive to firing rate. STTC is defined as

$$ STTC=\frac{1}{2}\left(\frac{P_A-{T}_B}{1-{P}_a{T}_B}+\frac{P_B-{T}_A}{1-{P}_B{T}_A}\right) $$

where T_A_ is the total recording time that lies within ±∆t of any spike from cell A. T_B_ is calculated similarly for cell B. P_A_ is the proportion of spikes from cell A which lie within ±∆t of any spike from cell B. P_B_ is calculated similarly for cell B. For our calculations ∆t was defined as 0.1 s. STTC is 1 with autocorrelation and − 1 when P_A_ = 0 and T_B_ = 1.

### Statistical analysis

All statistical analyses were carried out in Matlab (Mathworks, USA; RRID:SCR_001622). The sample sizes required for this study were estimated based on previous studies [22, 26]. For descriptive statistics, we used mean ± STD, or mean ± SEM where indicated. For box plots, the height of the boxes extended between the 25th and the 75th percentiles of the data. The horizontal bar and cross mark signified the median and mean, respectively. For plotting, outliers were defined as data points 1.5 times higher than or 1.5 times lower than the interquartile range and were shown as circles. The box plot whiskers extended to the most extreme data points that were not considered outliers. Outliers were not excluded from analyses. We considered *P* values less than 0.05 as significant. Significance values for comparisons of burst property means were calculated by fitting hierarchical linear mixed-effects models to cell data where the condition (monocular or binocular) and recording temperature (34 or 37 °C) were the fixed-effects, and recording/retina was the random-effect to correct for the non-independence of recorded cells. Significance values for comparisons of wave properties were also calculated by fitting linear mixed effects models as described above, except when comparing wave frequencies where the model only included terms for condition and temperature. In cases where samples were lognormal, we carried out a log transformation to bring samples to a normal distribution. In other cases, sample distributions had downward skews and were transformed with the exponential function. Comparing STTC values between enucleation conditions was similarly carried out using a hierarchical linear-mixed effects model where condition and temperature were fixed-effects, RGC pair distance was a covariate, and recording/retina was a random effect. STTC values were averaged for all RGC pairs by distance for each retina, resulting in a single value for each unique RGC pair distance. Before fitting the model, distance values were log transformed to improve linearity as shown in Fig. 6. All figures display data in their transformed state.

## Results

### Monocular enucleation has multiple effects on retinal waves

**Fig. 1:**
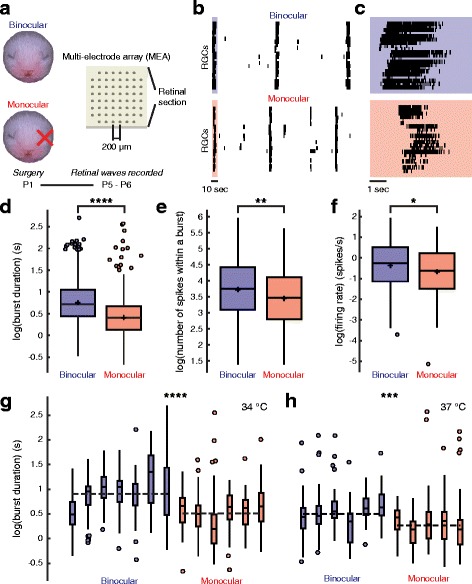
Effects of removing inter-eye competition on RGC activity. **a** An illustration of the enucleation protocol and MEA used for recording retinal waves. **b** Raster plots of wave activity from the retina of a binocular (**top**) and a monocular (**bottom**) ferret. **c** The RGC activity from highlighted periods shown in **b** on a finer time scale. **d**-**f**) Plots showing the difference in burst duration (s) (binocular, 2.39 ± 1.32; monocular, 1.67 ± 0.99; mean ± STD; T(2168) = 4.91, *P* = 9.9609 × 10^− 7^, linear mixed-effects model with log transformation) (**d**), number of spikes within a burst (binocular, 60.16 ± 53.26; monocular, 44.87 ± 39.82; mean ± STD; T(2168) = 2.92, *P* = 0.0036, linear mixed-effects model with log transformation) (**e**), and firing rate (spikes/s) (binocular, 1.19 ± 1.19; monocular, 0.86 ± 0.84; mean ± STD; T(2176) = 2.49, *P* = 0.0128, linear mixed-effects model with log transformation) (**f**), between RGCs from retinae of binocular or monocular ferrets. **g** - **h** Plots of RGC burst duration for all retinae recorded at a temperature of 34 °C (binocular, 2.76 ± 1.42; monocular, 1.82 ± 0.91; mean ± STD; T(1317) = 3.90, *P* = 9.952 × 10^− 5^, linear mixed-effects model with log transformation) (**g**) and 37 °C (binocular, 1.79 ± 0.83; monocular, 1.46 ± 1.07; mean ± STD; T(850) = 3.37, *P* = 0.00077, linear mixed-effects model with log transformation) (**h**). Dashed lines indicate group means. The heights of the box plots extend between the 25th and the 75th percentiles of the data. The horizontal bar and cross mark indicate the median and mean, respectively. Outliers are shown as circles and are data points that are 1.5 times higher than or 1.5 times lower than the interquartile range. The box plot whiskers extend to the most extreme data points that are not outliers. Binocular, *N* = 1178 cells, 13 retinae; Monocular, *N* = 1001 cells, 11 retinae. * = *P* < 0.05, ** = *P* < 0.01, *** = *P* < 0.001, **** = *P* < 0.0001

To investigate changes in the properties of retinal waves following the removal of a competing eye, we surgically removed one eye from newborn ferrets 1 day after birth (P1). Retinae were dissected away from the eyes of binocular and monocular ferrets between P5 and P6 and placed RGC layer side down on a 60-channel multielectrode array to record retinal wave activity ex vivo (Fig. 1a). We chose this time point as it has been previously shown that the expansion of the ipsilateral projection is retinal wave-dependent between P5 and P10 [8].

The waves recorded from the retinae of monocular and binocular ferrets appeared at first glance to be qualitatively similar (Fig. 1b). However, with further analysis, it was found that retinal waves were notably different in several ways following early monocular enucleation. The largest and most significant effects observed were those on RGC burst duration and the number of spikes within a burst (Fig. 1d-e). Compared to retinae from binocular ferrets, those from enucleates had RGCs whose bursts of action potentials were approximately 30% shorter in duration (binocular, 2.39 ± 1.32 s; monocular, 1.67 ± 0.99 s; mean ± STD; binocular, *N* = 1178 cells, 13 retinae; monocular, *N* = 1001 cells, 11 retinae; T(2168) = 4.91, *P* = 9.9609 × 10^− 7^, linear mixed-effects model with log transformation) (Fig. 1d). The reduction in burst duration was for the most part consistent across retinae recorded at temperatures of either 34 °C (binocular, 2.76 ± 1.42; monocular, 1.82 ± 0.91; mean ± STD; binocular, *N* = 731, 7 retinae; monocular, *N* = 592, 6 retinae; T(1317) = 3.90, *P* = 9.952 × 10^− 5^, linear mixed-effects model with log transformation) or 37 °C (binocular, 1.79 ± 0.83; monocular, 1.46 ± 1.07; mean ± STD; binocular, *N* = 447, 6 retinae; monocular, *N* = 409, 5 retinae; T(850) = 3.37, *P* = 0.00078, linear mixed-effects model with log transformation) (Fig. g-h). The impact on burst duration due to enucleation lead to the number of spikes within a burst to be reduced by approximately 25% (binocular, 60.16 ± 53.26; monocular, 44.87 ± 39.82; mean ± STD; binocular, *N* = 1178 cells, 13 retinae; monocular, *N* = 1001 cells, 11 retinae; T(2168) = 2.92, *P* = 0.00357, linear mixed-effects model with log transformation) (Fig. 1e). As expected, given that fewer spikes were contained within bursts, the overall firing rate of RGCs was reduced with enucleation (binocular, 1.19 ± 1.19 spikes/s; monocular, 0.86 ± 0.84 spikes/s; mean ± STD; binocular, *N* = 1178 cells, 13 retinae; monocular, *N* = 1001 cells, 11 retinae; T(2176) = 2.49, *P* = 0.0128, linear mixed-effects model with log transformation) (Fig. 1f).

**Fig. 2:**
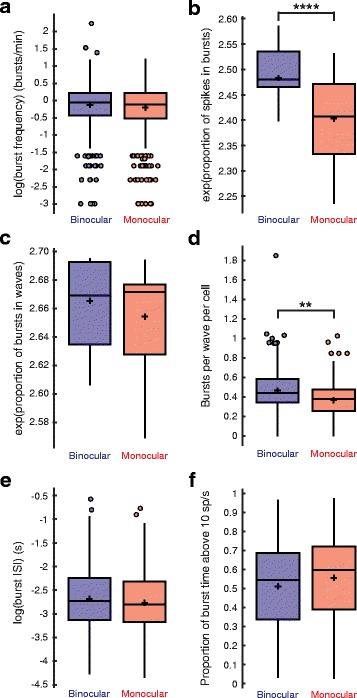
Other burst properties. **a** Burst frequency (bursts/min) (binocular, 1.02 ± 0.56; monocular, 0.96 ± 0.52; mean ± STD; T(2168) = 1.361, *P* = 0.174, linear mixed-effects model with log transformation), **b** proportion of spikes in bursts (binocular, 0.900 ± 0.155; monocular, 0.862 ± 0.186; mean ± STD; T(2168) = 5.44, *P* = 5.814 × 10^− 8^, linear mixed-effects model with exponential transformation), **c** proportion of bursts in waves (binocular, 0.978 ± 0.068; monocular, 0.975 ± 0.057; mean ± STD; T(2168) = 1.744, *P* = 0.0814, linear mixed-effects model with exponential transformation), **d** bursts per wave per cell (binocular, 0.467 ± 0.191; monocular, 0.371 ± 0.159; mean ± STD; T(2168) = 2.66, *P* = 0.0078, linear mixed-effects model), **e** ISI for all bursts (s) (binocular, 0.086 ± 0.062; monocular, 0.078 ± 0.055; mean ± STD; T(2168) = 0.981, *P* = 0.327, linear mixed-effects model with log transformation), and **f** proportion of burst time firing above 10 spikes/s for all bursts (binocular, 0.512 ± 0.217; monocular, 0.557 ± 0.213; mean ± STD; T(2168) = 1.783, *P* = 0.0747, linear mixed-effects model) for each condition. The heights of the box plots extend between the 25th and the 75th percentiles of the data. The horizontal bar and cross mark indicate the median and mean, respectively. Outliers are shown as circles and are data points that are 1.5 times higher than or 1.5 times lower than the interquartile range. The box plot whiskers extend to the most extreme data points that are not outliers. Binocular, *N* = 1178 cells, 13 retinae; Monocular, *N* = 1001 cells, 11 retinae. ** = *P* < 0.01, **** = *P* < 0.0001

**Fig. 3:**
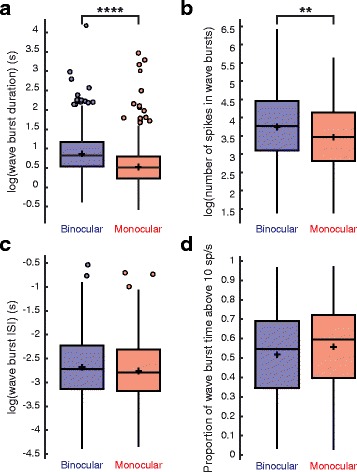
Wave burst properties. **a** Wave burst duration (s) (binocular, 2.51 ± 2.22; monocular, 1.77 ± 1.64; mean ± STD; T(2168) = 4.727, *P* = 2.426 × 10^− 6^, linear mixed-effects model with log transformation), **b** number of spikes within a wave burst (binocular, 62.23 ± 57.23; monocular, 46.21 ± 41.43; mean ± STD; T(2168) = 2.927, *P* = 0.0035, linear mixed-effects model with log transformation), **c** wave burst ISI (binocular, 0.084 ± 0.060; monocular, 0.077 ± 0.055; mean ± STD; T(2168) = 1.016, *P* = 0.310, linear mixed-effects model with log transformation), and **d** proportion of wave burst time firing above 10 spikes/s (binocular, 0.515 ± 0.217; monocular, 0.558 ± 0.212; mean ± STD; T(2168) = 1.799, *P* = 0.0722, linear mixed-effects model) for each condition. The heights of the box plots extend between the 25th and the 75th percentiles of the data. The horizontal bar and cross mark indicate the median and mean, respectively. Outliers are shown as circles and are data points that are 1.5 times higher than or 1.5 times lower than the interquartile range. The box plot whiskers extend to the most extreme data points that are not outliers. Binocular, *N* = 1178 cells, 13 retinae; Monocular, *N* = 1001 cells, 11 retinae. ** = *P* < 0.01, **** = *P* < 0.0001

In most other ways retinal waves were generally unaffected by monocular enucleation. Bursts occurred at the same frequency in both conditions (binocular, 1.02 ± 0.56; monocular, 0.96 ± 0.52; mean ± STD; binocular, *N* = 1178 cells, 13 retinae; monocular, *N* = 1001 cells, 11 retinae; T(2168) = 1.36, *P* = 0.174, linear mixed-effects model with log transformation) (Fig. 2a), and the vast majority of RGC spikes were contained within bursts, although this was slightly less so for the monocular condition (binocular, 0.900 ± 0.155 proportion of spikes in bursts; monocular, 0.862 ± 0.186 proportion of spikes in bursts; mean ± STD; binocular, *N* = 1178 cells, 13 retinae; monocular, *N* = 1001 cells, 11 retinae; T(2168) = 5.44, *P* = 5.814 × 10^− 8^, linear mixed-effects model with exponential transformation) (Fig. 2b). Bursts occurred almost exclusively during waves regardless of condition (binocular, 0.978 ± 0.068 proportion of bursts in waves; monocular, 0.975 ± 0.057 proportion of bursts in waves; mean ± STD; binocular, *N* = 1178 cells, 13 retinae; monocular, *N* = 1001 cells, 11 retinae; T(2168) = 1.74, *P* = 0.0814, linear mixed-effects model with exponential transformation) (Fig. 2c) but for the monocular condition, the number of bursts per wave per cell was slightly reduced (binocular, 0.467 ± 0.191; monocular, 0.371 ± 0.159; mean ± STD; binocular, *N* = 1178 cells, 13 retinae; monocular, *N* = 935 cells, 11 retinae; T(2168) = 2.66, *P* = 0.00777, linear mixed-effects model) (Fig. 2d). Additionally, the burst ISI (binocular, 0.086 ± 0.062 s; monocular, 0.078 ± 0.055 s; mean ± STD; binocular, *N* = 1178 cells, 13 retinae; monocular, *N* = 1001 cells, 11 retinae; T(2168) = 0.98, *P* = 0.327, linear mixed-effects model with log transformation) (Fig. 2e) and the proportion of burst time above 10 spikes/s (binocular, 0.512 ± 0.217; monocular, 0.557 ± 0.213; mean ± STD; binocular, *N* = 1178 cells, 13 retinae; monocular *N* = 935 cells, 11 retinae; T(2168) = 1.78, *P* = 0.0747, linear mixed-effects model) (Fig. 2f) were unchanged with enucleation, further confirming that the reduction in the number of spikes in a burst was due to shorter burst durations. When analyses were constrained to only wave associated bursts, the differences between conditions were consistent with our general findings (Fig. 3a-d).

**Fig. 4:**
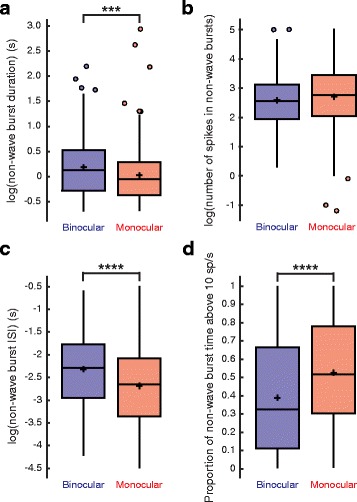
Non-wave burst properties. **a** Non-wave burst duration (s) (binocular, 1.46 ± 1.11; monocular, 1.26 ± 1.52; mean ± STD; T(514) = 3.35, *P* = 0.00086, linear mixed-effects model with log transformation), **b** number of spikes within a non-wave burst (binocular, 20.02 ± 22.19; monocular, 24.63 ± 26.32; mean ± STD; T(514) = 1.078, *P* = 0.282, linear-mixed effects model with log transformation), **c** non-wave burst ISI (binocular, 0.137 ± 0.117; monocular, 0.098 ± 0.093; mean ± STD; T(514) = 5.04, *P* = 6.534 × 10^− 7^, linear mixed-effects model with log transformation), and **d** proportion of non-wave burst time firing above 10 spikes/s (binocular, 0.390 ± 0.291; monocular, 0.527 ± 0.290; mean ± STD; T(514) = 5.64, *P* = 3.298 × 10^− 8^, linear mixed-effects model) for each condition. The heights of the box plots extend between the 25th and the 75th percentiles of the data. The horizontal bar and cross mark indicate the median and mean, respectively. Outliers are shown as circles and are data points that are 1.5 times higher than or 1.5 times lower than the interquartile range. The box plot whiskers extend to the most extreme data points that are not outliers. Binocular, *N* = 243 cells, 13 retinae. Monocular, *N* = 268 cells, 11 retinae. *** = *P* < 0.001, **** = *P* < 0.0001

Other observed effects on burst properties due to enucleation were unique to non-wave bursts, which were very rare in both conditions (Fig. 2c). Non-wave bursts, like wave bursts, were shorter in duration in the monocular condition (binocular, 1.46 ± 1.11 s; monocular, 1.26 ± 1.52 s; mean ± STD; binocular, *N* = 245 cells, 13 retinae. Monocular, *N* = 272 cells, 11 retinae; T(514) = 3.35, *P* = 0.00086, linear mixed-effects model with log transformation) (Fig. 4a), but had shorter burst ISIs (binocular, 0.137 ± 0.117 s; monocular, 0.098 ± 0.093 s; mean ± STD; binocular, *N* = 245 cells, 13 retinae. Monocular, *N* = 272 cells, 11 retinae; T(514) = 5.04, *P* = 6.534 × 10^− 7^, linear mixed-effects model with log transformation) (Fig. 4c) and spent a larger proportion of time firing at rates above 10 spikes/s (binocular, 0.390 ± 0.291; monocular, 0.527 ± 0.290; mean ± STD; binocular, *N* = 245 cells, 13 retinae. Monocular, *N* = 272 cells, 11 retinae; T(514) = 5.65, *P* = 3.298 × 10^− 8^, linear mixed-effects model) (Fig. 4d). These combined effects resulted in non-wave bursts containing a similar number of spikes in both conditions (binocular, 20.02 ± 22.20; monocular, 24.63 ± 26.32; mean ± STD; binocular, *N* = 245 cells, 13 retinae. Monocular, *N* = 272 cells, 11 retinae; T(514) = 1.08, *P* = 0.282, linear-mixed effects model with log transformation) (Fig. 4b).

**Fig. 5:**
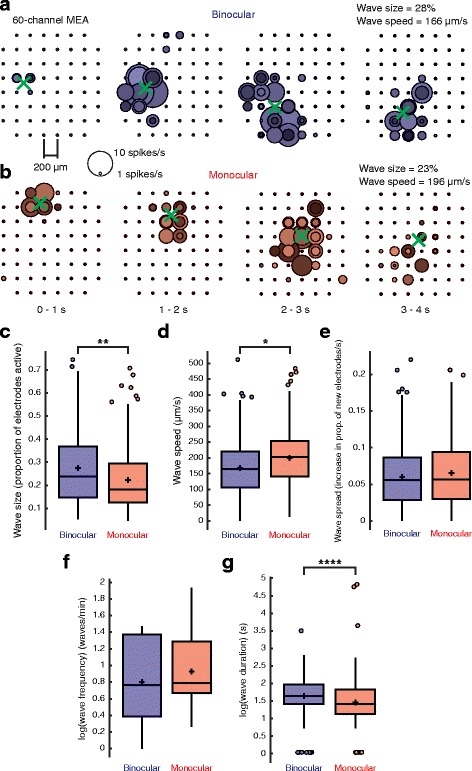
Effects of removing inter-eye competition on the spatiotemporal properties of retinal waves. **a**-**b**) Visualizations of representative waves recorded from the retina of a binocular (**a**) or and a monocular (**b**) ferret. Each colored circle represents an RGC active during the period designated on the bottom of (**b**). Circle size corresponds to RGC firing rate as shown by the legend between (**a**) and (**b**). The green cross indicates the center of the wave for each period. **c**-**g** Plots showing the difference in size (proportion of electrodes active) (binocular, 0.275 ± 0.158; monocular, 0.224 ± 0.130; mean ± STD; T(977) = 2.58, *P* = 0.00999, linear mixed-effects model) (**c**), speed (μm/s) (binocular, 167.67 ± 77.63; monocular, 199.39 ± 83.63; mean ± STD; T(855) = 2.22, *P* = 0.0267, linear mixed-effects model) (**d**), spread (proportion of new electrodes active/s) (binocular, 0.060 ± 0.042; monocular, 0.065 ± 0.045; mean ± STD; T(855) = 0.821, *P* = 0.412, linear mixed-effects model) (**e**), frequency (waves/min) (binocular, 2.53 ± 1.26; monocular, 2.86 ± 1.64; mean ± STD; T(21) = 1.216, *P* = 0.237, linear mixed-effects model with log transformation) (**f**), and duration (s) (binocular, 5.79 ± 3.15; monocular, 5.07 ± 7.60; mean ± STD; T(977) = 5.74, *P* = 1.233 × 10^− 8^, linear mixed-effects model with log transformation) (**g**) of waves recorded from the retinae of binocular and monocular ferrets. The heights of the box plots extend between the 25th and the 75th percentiles of the data. The horizontal bar and cross mark indicate the median and mean, respectively. Outliers are shown as circles and are data points that are 1.5 times higher than or 1.5 times lower than the interquartile range. The box plot whiskers extend to the most extreme data points that are not outliers. Binocular, *N* = 451 waves, 13 retinae. Monocular, *N* = 408 waves, 11 retinae. * = *P* < 0.05, ** = *P* < 0.01, **** = *P* < 0.0001

Lastly, we found small but significant effects on retinal wave size (binocular, 0.275 ± 0.158 proportion of electrodes active; monocular, 0.224 ± 0.130 proportion of electrodes active; mean ± STD; binocular, *N* = 495 waves, 13 retinae. Monocular, *N* = 485 waves, 11 retinae; T(977) = 2.58, *P* = 0.00999, linear mixed-effects model) (Fig. 5c) and speed (binocular, 167.67 ± 77.63 μm/s; monocular, 199.39 ± 83.63 μm/s; mean ± STD; binocular, *N* = 449 waves, 13 retinae. Monocular, *N* = 409 waves, 11 retinae; T(855) = 2.22, *P* = 0.0267, linear mixed-effects model) (Fig. 5d). However, the rate that waves spread to new electrodes was unchanged with enucleation (binocular, 0.060 ± 0.042 proportion of new electrodes active/s; monocular, 0.065 ± 0.045 proportion of new electrodes active/s; mean ± STD; binocular, *N* = 449 waves, 13 retinae. Monocular, *N* = 409 waves, 11 retinae; T(855) = 0.821, *P* = 0.412, linear mixed-effects model) (Fig. 5e). Wave frequency was not significantly different with enucleation (binocular, 2.53 ± 1.26; monocular, 2.86 ± 1.64; mean ± STD; binocular, *N* = 13 retinae; monocular, *N* = 11 retinae; T(21) = 1.216, *P* = 0.237, linear-mixed effects model) (Fig. 5f). However, there was a shortening of wave duration (binocular, 5.79 ± 3.15 s; monocular, 5.07 ± 7.60 s; mean ± STD; binocular, *N* = 495 waves, 13 retinae. Monocular, *N* = 485 waves, 11 retinae; T(977) = 5.744, *P* = 1.233 × 10^− 8^, linear mixed-effects model with log transformation) (Fig. 5g) consistent with the shortening of RGC burst duration.

### Shortening RGC burst duration reduces pairwise RGC correlation levels

**Fig. 6:**
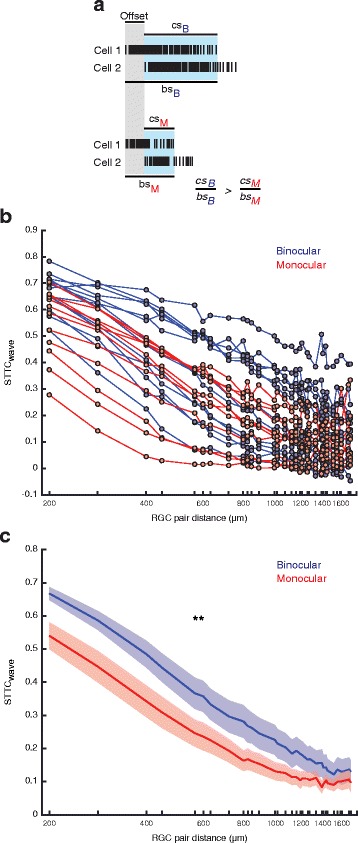
Removing inter-eye competition reduces levels of pairwise RGC correlation during waves*.* **a** Illustration of the hypothesized effects of shortening burst duration on pairwise RGC correlation levels during waves. The gray shading is the offset time between bursts due to the propagation speed of a retinal wave. bs = burst spikes. cs = correlated spikes. Subscripts M and B signify monocular or binocular spikes. **b** Plot of STTC over distance for all retinae recorded. **c** Plot of average STTC as a function of RGC pair distance for both conditions (T(738) = 2.85, *P* = 0.00452, linear mixed-effects model). Tick marks indicate cell pair distances included for STTC measures as constrained by the spatial organization of the MEA. Binocular, *N* = 401 distances, 13 retinae. Monocular, *N* = 341 distances, 11 retinae. Error bars are SEM. ** = *P* < 0.01

The effects on RGC bursts were intriguing, as bursts appear to play a critical role in retinofugal refinement [9, 17, 28, 29]. Due to the spatiotemporal properties of retinal waves, the bursting activity of neighboring RGCs is highly correlated but is largely uncorrelated between pairs of RGCs that are distant from each other [26, 30]. A large body of work has supported the hypothesis that correlated activity is critical for retinotopic refinement of RGC afferent terminals within the dLGN and superior colliculus [9, 26, 28, 31]. If the duration of bursts is shortened, the pairwise correlation between RGCs with offset burst times, as is the case during propagating waves, should be reduced (Fig. 6a).

Based on the hypothesis that a shortening of burst duration should reduce pairwise correlation levels between RGCs during waves (Fig. 6a), we carried out additional measurements to determine if levels of correlated activity decreased with enucleation. To quantify pairwise RGC correlations we calculated the “spike time tiling coefficient” (STTC) [27], which is a pairwise correlation measure that is bounded and insensitive to firing rate. Retinae from enucleates and binocular ferrets showed pairwise RGC correlations that fell as a function of the distance between cell pairs consistent with what has been previously described [30] (Fig. 6b). However, as predicted, levels of correlated RGC activity during waves were reduced across RGC pair distances for retinae from enucleates (binocular, *N* = 401 distances, 13 retinae; monocular, *N* = 341 distances, 11 retinae; T(738) = 2.85, *P* = 0.00452, linear mixed-effects model) (Fig. 6c).

**Fig. 7:**
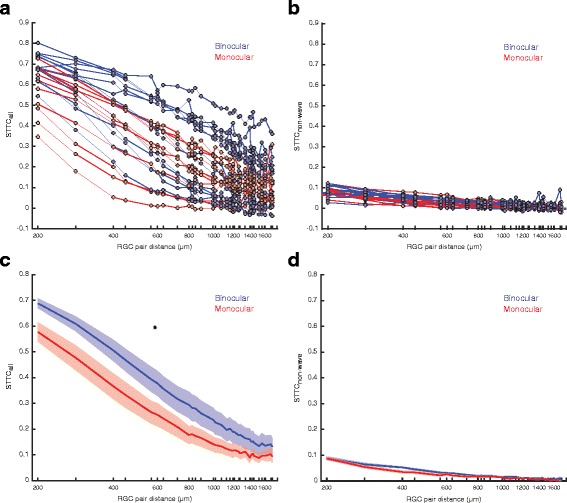
Effect of enucleation on levels of pairwise RGC correlation for all activity and non-wave activity. Plots of STTC over distance for all retinae recorded for (**a**) all RGC activity and (**b**) non-wave activity. Plots of average STTC as a function of RGC pair distance for both conditions for (**c**) all recorded activity (T(738) = 2.56, *P* = 0.0105, linear mixed-effects model) and (**d**) non-wave activity (T(738) = 1.02, *P* = 0.310, linear-mixed effects model). Tick marks indicate cell pair distances included for STTC measures as constrained by the spatial organization of the MEA. Binocular, *N* = 401 distances, 13 retinae. Monocular, *N* = 341 distances, 11 retinae. Error bars are SEM. * = *P* < 0.05

The number of spikes within bursts and non-wave burst properties between retinae from binocular and monocular ferrets were different as described above. Although spikes outside bursts and non-wave bursts were rare, it may be the case that they had an impact on overall levels of correlated activity. For non-wave associated activity, pairwise RGC correlation levels were not significantly different between conditions (binocular, *N* = 401 distances, 13 retinae; monocular, *N* = 341 distances, 11 retinae; T(738) = 1.02, *P* = 0.310, linear-mixed effects model) (Fig. 7b and d). However, when correlation levels were measured for all recorded RGC activity, a difference between conditions was still found (binocular, *N* = 401 distances, 13 retinae; monocular, *N* = 341 distances, 11 retinae; T(738) = 2.56, *P* = 0.0106, linear mixed-effects model) (Fig. 7a and c) indicating that correlation levels are predominately determined by the properties of wave bursts.

## Discussion

We have previously shown that retinal waves are critical for the targeting of retinogeniculate afferents following the removal of competing inputs to the dLGN [8]. While this work demonstrated that aspects of afferent terminal targeting during retinogeniculate refinement are retinal wave-dependent, it remained unclear what retinal wave properties might be necessary for this process. This study aimed to elucidate what retinal wave properties could dynamically guide afferent terminal targeting when an eye is lost. We show that the removal of the competing eye alters the duration of retinal wave associated RGC bursts, which has impacts on RGC correlation. Since studies have shown that correlation plays a critical role in retinogeniculate refinement, our finding is consistent with the hypothesis that retinal wave activity can dynamically guide retinogeniculate refinement while taking into consideration the presence of inter-eye competition.

We should note that since monocular enucleation is the complete removal of an organ, there is essentially no sham surgery that can fully replicate its potential side effects. Thus, our study cannot authoritatively rule out effects on retinal waves due to stress the ferrets may have experienced due to the monocular enucleation procedure. However, there is data to suggest that noxious stimuli at this age are unlikely to have large impacts on the brain. Studies have shown that newborn mammals that undergo extended periods of brain development *ex utero* are hyporesponsive to noxious stimuli in the first two postnatal weeks [32]. Additionally, we found that newborn ferrets that underwent the short enucleation procedure healed quickly and did not display any developmental stunting or signs of distress. Ultimately, future experiments utilizing more targeted interventions are required to elucidate further how monocular enucleation affects retinal wave activity.

### The role of burst duration and RGC correlation

**Fig. 8:**
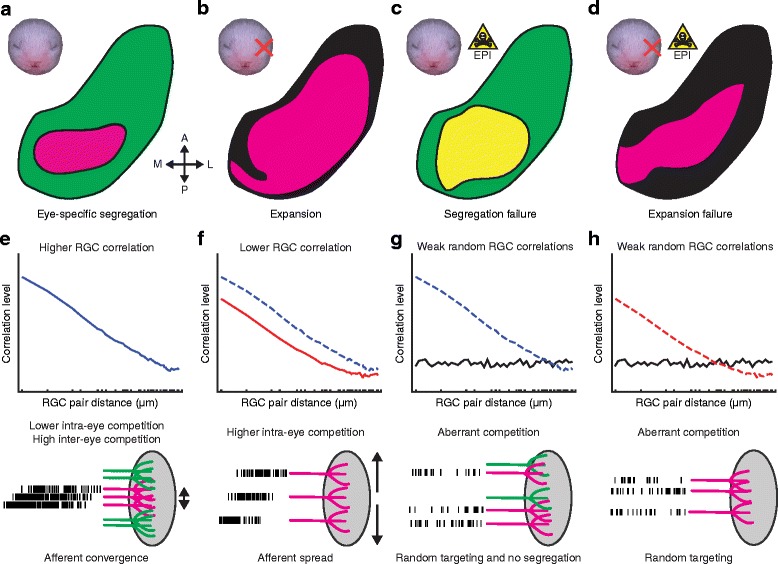
A model to explain the relationship between pairwise RGC correlation levels and retinogeniculate refinement. **a** - **d** Illustrations of horizontal sections at P10 with retinogeniculate laminae shaded. Ipsilateral inputs are shown in magenta; contralateral inputs are shown in green. A = anterior, P = posterior, M = medial, L = lateral. In binocular ferrets, the ipsilateral projection is condensed and segregated from the contralateral projection by P10 (**a**). In monocular ferrets, the surviving ipsilateral projection is greatly expanded following the elimination of contralateral input (**b**). In binocular ferrets when retinal waves are blocked, eye-specific segregation fails and afferent targeting is abnormally expanded (**c**). In monocular ferrets when retinal waves are blocked, expansion of the ipsilateral projection is disrupted (**d**). **e** Binocular ferrets have longer RGC bursts that result in higher pairwise RGC correlation levels. Higher levels of correlated RGC activity decreases intra-eye competition, which can better facilitate the formation of eye-specific laminae during inter-eye competition. **f** Monocular ferrets have shortened RGC bursts that result in lower pairwise RGC correlation levels. The reduction in pairwise RGC correlations increases intra-eye competition, resulting in afferent spread and expanded ipsilateral laminae. Blue dashed line represents RGC correlation levels for the binocular condition. **g**-**h** EPI treatment decorrelates RGC activity and disrupts intra-eye and inter-eye competition, resulting in random afferent targeting and similar ipsilateral projection size in EPI-treated binocular and monocular ferrets. Blue and red dashed lines represent RGC correlation levels for the untreated binocular and monocular conditions respectively

RGC bursts are important for refinement of the retinogeniculate pathway [4, 9, 17, 28, 29]. Thus, the changes in RGC burst duration following monocular enucleation (Fig. 1) may indicate a role of the competing eye in influencing the refinement of the retinogeniculate pathway of the other. Additionally, we found that RGC burst duration scales RGC correlation (Fig. 6). Removal of an eye thus results in reductions in correlation level that are associated with the expansive targeting seen in the dLGN following monocular enucleation [8]. Since a large body of experimental and theoretical work has supported a role for pairwise RGC correlations in retinogeniculate refinement [8, 9, 18, 26, 28, 31, 33, 34], we believe this finding is unlikely to be coincidental. Based on the evidence that afferent terminal targeting is guided by competition [8], we propose a model where RGC burst duration scales RGC correlation to dynamically guide afferent targeting within the dLGN during visual system development:

In binocular ferrets, RGC burst duration is longer, which results in higher pairwise RGC correlation levels within an eye. Higher RGC correlation levels decrease intra-eye competition, which in the context of inter-eye competition between ipsilateral and contralateral inputs, is optimal for establishing eye-specific laminae (Fig. 8a and e). Conversely, in monocular ferrets, burst durations are shorter, RGC correlations are reduced, and intra-eye competition is increased. Increasing intra-eye competition facilitates the spread of afferents resulting in expanded ipsilateral laminae, thus utilizing more synaptic space within the dLGN when contralateral afferents from the competing eye are absent (Fig. 8b and f). Previous studies have effectively blocked retinal waves (i.e., spatiotemporal correlations) in ferrets by decorrelating the activity of neighboring RGCs with the cholinergic agonist epibatidine (EPI) [7, 8, 35]. Blocking retinal waves with EPI in binocular ferrets disrupts eye-specific segregation and lamination, and in enucleates, the lamination and expansion of retinogeniculate projections, resulting in ipsilateral projections of approximately the same size in binocular and monocular ferrets [8] (Fig. 8c-d). Thus, blocking retinal waves with EPI results in abnormal afferent competition in both the binocular and monocular condition, causing randomized afferent terminal targeting that is no longer being effectively guided by intra-eye and inter-eye competition (Fig. 8g-h).

Consistent with our model, the importance of low intra-eye competition for eye-specific segregation in binocular ferrets was recently demonstrated [36]. In this study, we used an immunotoxin to ablate starburst amacrine cells (SACs) that are responsible for retinal wave generation. This SAC ablation resulted in a reduction of RGC correlation similar to that seen following monocular enucleation, with fewer SACs leading to less RGC correlation. In binocular ferrets where laminae appeared normal, SAC ablation levels were symmetric across eyes. However, in ferrets where one eye’s retinogeniculate projection was larger, SAC ablation was lower in that eye (i.e., SAC ablation was asymmetric). This result demonstrated that when intra-eye competition is increased in one eye relative to the other due to asymmetric SAC ablation, the eye with increased intra-eye competition (less RGC correlation) is hindered in its ability to compete for synaptic space in the dLGN and loses territory to the eye with lower intra-eye competition (more RGC correlation). Similarly, recent studies in mice have used transgenic lines to investigate the role of retinal wave size in retinogeniculate refinement. In two different transgenic mouse lines, neighboring RGC correlation levels were reduced but not eliminated [33, 34]. Consistent with our model, these studies showed that reduced RGC correlation disrupted eye-specific segregation [33, 34] and resulted in an expanded ipsilateral projection in binocular transgenic mice [33]. Additionally, in transgenic enucleates where competing contralateral inputs were absent, fine-scale retinogeniculate refinement appeared normal [33]. We must note, however, that while the above studies demonstrated the importance high RGC correlation levels for the establishment of eye-specific laminae, the nature of the effects for ferrets and mice were different. In ferrets, moderate increases in relative intra-eye competition shrank eye-specific laminae and had minor effects on eye-specific segregation [36], while in transgenic mice ipsilateral projection size increased for both eyes and eye-specific segregation was disrupted [33, 34]. The difference can be explained by the high levels of RGC correlation found in ferrets relative to mice [35]. The reduction to the mouse’s already relatively low RGC correlation levels in the above transgenic lines may have prevented effective inter-eye competition [33, 34] and resulted in expanded ipsilateral projections [33] like observed in monocular ferrets where no inter-eye competition is present [8, 37], or in binocular ferrets when EPI treatment completely blocks retinal waves [7, 8].

Surprisingly, bursts that occurred outside of waves were affected differently by monocular enucleation. For non-wave bursts, burst ISIs were shorter and the percentage of burst time above 10 spikes/s was greater (Fig. 4). However, it is important to note that non-wave bursts made up less than 3% of all bursts in either condition (Fig. 2) and do not appear to have any significant impact on overall levels of pairwise RGC correlation (Fig. 7). For these reasons, the observed changes to non-wave burst properties are unlikely to be related to the altered retinogeniculate refinement in enucleates. It is hard to speculate on why these effects are observed for non-wave bursts and not wave bursts, but after additional studies are carried out, it may prove valuable in understanding the neurobiological mechanisms by which monocular enucleation alters burst properties.

### A signal for the presence of the competing eye?

Removing an eye alters retinal waves in the one that is spared. However, this study is unable to elucidate the neurobiological mechanism underlying such effects. It is important to note that we observe differences in retinal waves due to monocular enucleation ex vivo, indicating that the competing eye must be inducing relatively long-lasting effects in the opposing retina. One candidate mechanism is inputs from the competing eye onto neuromodulator releasing amacrine cells, which modify synaptic connectivity or other cell membrane properties [38]. Retino-retinal projecting retinal ganglion cells (rrRGCs) have been identified in several vertebrate species [39–41] and are greatest in number during early visual system development in rodents [39]. While a direct projection between the retinae to signal the presence of a competing eye is perhaps the most parsimonious explanation for the effects on retinal waves reported here, there is no direct evidence that rrRGCs modulate retinal wave activity. Future experiments to target these cells carefully, with either ablation or silencing, will be necessary to understand their role, if any, in visual system development.

## Conclusion

Our results demonstrate a novel phenomenon whereby the removal of a competing region of the central nervous system influences the patterned spontaneous neural activity of another. When the competing eye is absent, it shortens the RGC burst duration of the surviving eye. This effect on RGC burst duration scales the levels of RGC correlation in the developing retina, and reduced correlation levels coincide with the retinal wave-dependent spread of the retinogeniculate projection following the loss of an eye. Based on these novel findings and their association with the retinal-wave-dependent anatomical remodeling found in enucleates, we propose the hypothesis that the presence or absence of the competing eye dynamically scales afferent competition to guide retinogeniculate refinement during visual system development.

## Abbreviations

dLGN: Dorsal lateral geniculate nucleus
EPI: Epibatidine
ISI: Inter-spike interval
MEA: Multi-electrode array
RGC: Retinal ganglion cell
rrRGC: Retino-retinal projecting retinal ganglion cell
SAC: Starburst amacrine cell
STTC: Spike time tiling coefficient
